# Restoration of the Ellipsoid Zone and Visual Prognosis at 1 Year after Surgical Macular Hole Closure

**DOI:** 10.1155/2016/1769794

**Published:** 2016-01-28

**Authors:** Hiruma Hasebe, Naoki Matsuoka, Hiroko Terashima, Ryo Sasaki, Eriko Ueda, Takeo Fukuchi

**Affiliations:** Division of Ophthalmology and Visual Science, Niigata University, 1-757 Asahimachi-dori, Chuo-ku, Niigata City, Niigata 9518510, Japan

## Abstract

*Purpose*. To evaluate the restoration of the ellipsoid zone (EZ) and its influence on visual prognosis 1 year after surgical macular hole (MH) closure.* Method*. Subjects were patients with stage 2, 3, or 4 idiopathic MH who underwent primary vitrectomy that resulted in successful hole closure. Nineteen eyes with both EZ disruption with foveal detachment and a continuous external limiting membrane on optical coherence tomography during the early postoperative period were included in this study.* Result*. EZ disruption was restored in 10 eyes (53%, Group A) and remained in 9 eyes (47%, Group B) at 1 year after surgery. In Group B, the diameter of the residual EZ disruption was 54.7 ± 33.1 *μ*m. LogMAR visual acuity (VA) 1 year after surgery was significantly better than preoperative VA in each group (Group A: −0.007 ± 0.102; *P* < 0.001; Group B: 0.051 ± 0.148; *P* < 0.001), but there was no significant difference between the 2 groups (*P* = 0.332). There was no significant correlation between logMAR VA and EZ disruption diameter at 1 year after surgery.* Conclusion*. EZ was restored in 53% of eyes at 1 year after surgical closure of idiopathic MH. Mean residual EZ disruption diameter was 54.7 ± 33.1 *μ*m. Neither resolved nor residual EZ disruption influenced postoperative VA.

## 1. Introduction

Surgical closure of a macular hole (MH) was first reported by Kelly and Wendel [1]. With advances in instrument development and vitreous surgical techniques, the outcome of this treatment has improved remarkably [2–4]. These days, the postoperative hole closure rate is over 90% [5, 6]. Recent studies using high-resolution optical coherence tomography (OCT) have revealed that reconstruction of the outer layer of the retina is necessary for good visual prognosis after surgical MH closure [7–14]. The ellipsoid zone (EZ) appears disrupted on postoperative OCT images, but visual improvement occurs as the EZ is gradually restored [11, 12]. However, details of EZ disruption and restoration during the late postoperative period are not yet fully understood. Only a few studies have reported on the restoration of EZ disruption 1 year after successful MH closure [9, 10, 15, 16], but the closure rate, the size of the remaining EZ disruption, and those influences on visual prognosis were not shown in detail in these studies.

In this study, we investigated the status of the EZ on OCT 1 year after surgery in eyes with surgically closed idiopathic MH and evaluated the restoration rate of the EZ disruption and the diameter of the remaining EZ disruption. We analyzed differences in visual acuity (VA) associated with these changes in the EZ during the year after surgery.

## 2. Patients and Methods

**Figure 1:**
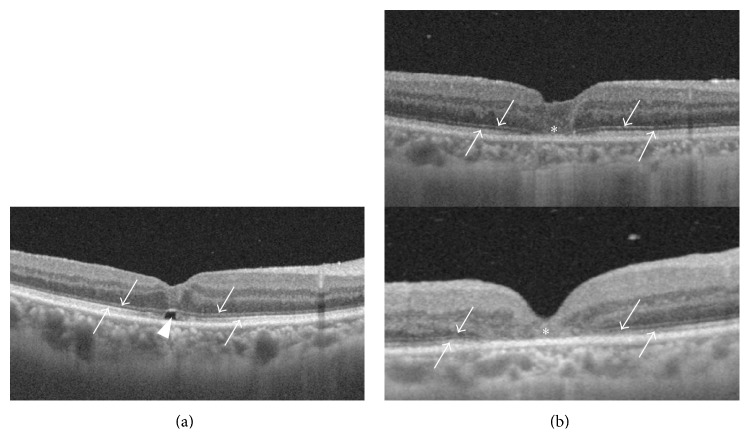
Macular OCT images of a patient who was included in the study (a) and excluded from the study (b). (a) The ellipsoid zone (EZ; up arrow) is disrupted with foveal detachment (arrowhead) beneath a continuous external limiting membrane (ELM; down arrow). (b) Both the EZ (up arrow) and the ELM (down arrow) are disrupted at the fovea (asterisk). Foveal detachment is not observed.

We reviewed the medical records of patients with idiopathic MH who underwent successful MH closure via primary vitrectomy at Niigata University Hospital from January 2013 to December 2014. This study followed the tenets of the Declaration of Helsinki. Subjects who met the following criteria were included in this study: stage 2, 3, or 4 MH; postoperative follow-up duration of more than 1 year; swept-source OCT (SS-OCT) which was used for postoperative examinations; and both EZ disruption with foveal detachment and a continuous external limiting membrane (ELM) which were found on OCT during the early postoperative period (within 10 days) (Figure 1(a)). We excluded eyes with stage 1 MH, pathological macular changes, and MH that was not successfully closed via primary vitrectomy. We also excluded eyes with both EZ and ELM disruption on OCT during the early postoperative period (Figure 1(b)).

There were 19 eyes in 18 patients included in the study. The patients consisted of 15 females and 3 males, aged 51–79 (mean ± SD, 63.8 ± 6.2) years. Preoperative symptom duration ranged from 1 to 9 (2.95 ± 1.90) months. There were 6, 7, and 6 eyes with stage 2, 3, and 4 holes, respectively (Table 1). No eyes showed restoration of the EZ on OCT during the early postoperative period.

All of the patients underwent 3-port pars plana vitrectomy using a 25-gauge system and simultaneous cataract surgery with intraocular lens implantation under local anesthesia. Vitrectomy consisted of core vitrectomy, intravitreal injection of triamcinolone acetonide (TA), surgical vitreous detachment in eyes with stage 2 and 3 MH, and peeling of the internal limiting membrane (ILM) around the hole. TA or 0.25% indocyanine green was used to visualize the ILM. Ten percent SF6 gas tamponade was used and patients were instructed to maintain a facedown position for 4 to 6 days postoperatively. All patients received a detailed explanation of the treatment and provided signed informed consent for the surgical procedure before surgery.

We obtained pre- and postoperative macular OCT images using SS-OCT (DRI-1 Atlantis, Topcon, Tokyo, Japan), which provided 100,000 A-scans/sec with an axial resolution of 8 *μ*m and a transverse resolution of 20 *μ*m. Radial scans in 12 directions and a 3D-scan in a 6 mm square area centered on the macula were obtained. Each radial scan had 1024 axial scans with a length of 12 mm and the 3D-scan consisted of 256 horizontal lines with 512 axial scans in each. The interval between scan lines in the 3D-scan was 23.44 *μ*m ( = 6 mm/256 lines). We used 3D-scan images to evaluate whether EZ was restored or not. The diameter of the EZ disruption was calculated by multiplying 23.44 *μ*m by the number of 3D-scan images on which EZ disruption was found in the macula. Preoperative MH diameter was measured in the same way. If 3D-scan had not been performed preoperatively, we measured preoperative radial scan images manually to determine the largest width between the edges of the hole as the MH diameter.

Subjects were classified into 2 groups by EZ status at the 1-year postoperative visit. Group A consisted of eyes in which EZ was restored and Group B consisted of eyes in which EZ disruption remained. The diameter of the residual EZ disruption was analyzed in Group B. The best corrected visual acuity (BCVA) was converted to logMAR units for statistical analysis. Correlations between logMAR VA and EZ disruption diameter were analyzed. Pre- and postoperative logMAR VA, age, symptom duration, preoperative MH diameter, and number of eyes with stage 4 MH in the 2 groups were compared.

## 3. Results

**Figure 2:**
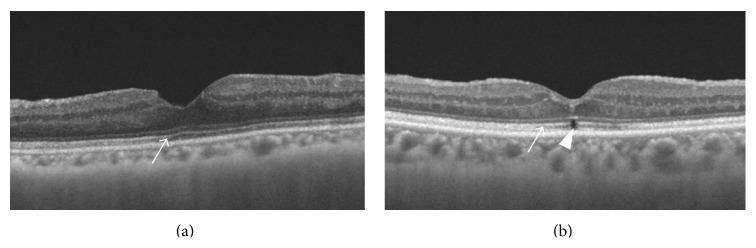
Macular OCT images of subjects in Groups A (a) and B (b). (a) The EZ (arrow) has been restored and continuous. (b) The EZ disruption (arrowhead) remains.

EZ was restored in 10 of 19 eyes (53%) (Group A, Figure 2(a)) and remained in 9 eyes (47%) (Group B, Figure 2(b)) at 1-year postoperative visit (Table 1). In Group A, EZ was restored in 1 eye (10%) at 1 month, 4 eyes (40%) at 3 months, 5 eyes (50%) at 6 months, and 10 eyes (100%) at 9 months postoperatively. The mean diameter of EZ disruption was significantly smaller in Group A than Group B at 1 month (Group A 96.2 ± 65.9 and Group B 234.4 ± 125.5 *μ*m, *P* = 0.009), 3 months (38.0 ± 57.5 and 125.0 ± 99.1 *μ*m, *P* = 0.041), and 6 months (25.8 ± 37.4 and 89.9 ± 74.7 *μ*m, *P* = 0.037) (Student's *t*-test, resp.). In Group B, the diameter of the remaining EZ disruption at 1 year ranged from 23.4 to 140.6 *μ*m and the mean diameter was 54.7 ± 33.1 *μ*m. Only 1 eye had an EZ disruption with a diameter greater than 100 *μ*m (Table 2).

Preoperative logMAR VA was not significantly different between the 2 groups (Group A, 0.592 ± 0.243, and Group B, 0.610 ± 0.343; *P* = 0.894; Student's *t*-test). LogMAR VA at 1 month, 3 months, 6 months, and 1 year after surgery was significantly better than preoperative VA in each group (Group A, 0.188 ± 0.246, *P* < 0.001; 0.078 ± 0.172, *P* < 0.001; 0.061 ± 0.146, *P* < 0.001; −0.007 ± 0.102, *P* < 0.001; Group B, 0.255 ± 0.183, *P* = 0.019; 0.137 ± 0.144, *P* = 0.002; 0.083 ± 0.128, *P* = 0.003; 0.051 ± 0.148, *P* < 0.001; paired *t*-test, resp.) but was not significantly different between the 2 groups at each period (*P* = 0.527, *P* = 0.451, *P* = 0.750, and *P* = 0.332; Student's *t*-test, resp.) (Table 3).

**Figure 3:**
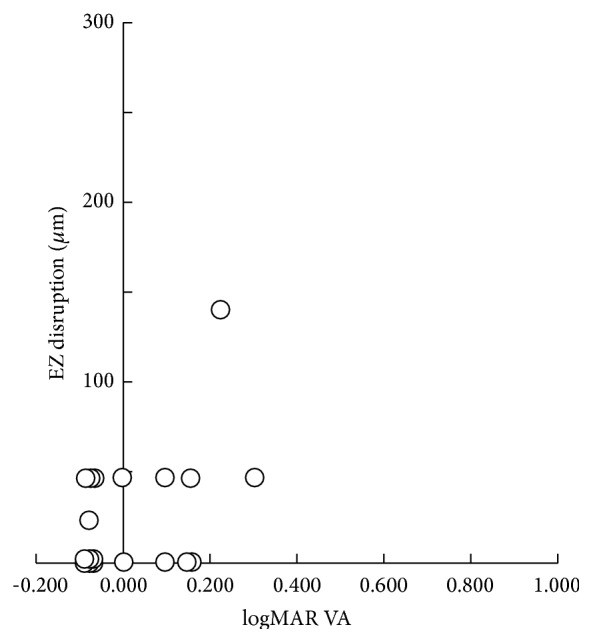
Scatter plot of logMAR VA and diameter of the EZ disruption at the 1-year postoperative visit. There was no significant correlation (*r*_*s*_ = 0.307, *P* = 0.201; Spearman's correlation coefficient test).

Mean logMAR VA of all subjects was significantly improved during the 1-year postoperative period (preoperative 0.600 ± 0.286, 0.218 ± 0.217 at 1 month, 0.104 ± 0.158 at 3 months, 0.070 ± 0.135 at 6 months, and 0.021 ± 0.126 at 1 year; all *P* < 0.001, paired *t*-test). There was no significant correlation between logMAR VA and EZ disruption diameter at 1 month, 3 months, 6 months, and 1 year after surgery (*r*_*s*_ = −0.228, *P* = 0.395; *r*_*s*_ = −0.169, *P* = 0.530; *r*_*s*_ = −0.033, *P* = 0.902; *r*_*s*_ = 0.307, *P* = 0.201; Spearman's rank correlation coefficient test, resp.) (Table 3, Figure 3).

Patient age (Group A, 65.1 ± 7.6, and Group B, 62.4 ± 4.4 years), symptom duration (Group A, 3.10 ± 2.42, and Group B, 2.78 ± 1.20 months), preoperative MH diameter (Group A, 307.0 ± 155.0, and Group B 370.4 ± 188.5 *μ*m), and number of eyes with stage 4 MH (Group A, 1 eye, and Group B, 5 eyes) were not significantly different between the 2 groups (*P* = 0.377, *P* = 0.723, and *P* = 0.432; Student's *t*-test; *P* = 0.057; Fisher's exact probability test, resp.) (Table 1).

## 4. Discussion

Recent studies using high-resolution OCT have revealed abnormalities of the outer layer of the retina even in eyes with surgically closed MH that undergo a process of reconstruction [7, 8, 11–14, 17]. Reconstruction of the foveal ELM precedes restoration of the foveal photoreceptor layer and EZ [11, 12]. Restoration of the EZ is important for postoperative VA improvement. Visual outcomes after MH closure are significantly better in eyes with restored EZ than in those with disrupted EZ [7, 8, 11]. However, these studies evaluated the restoration of EZ disruption within 6 months of surgery. EZ restoration or disruption during the late postoperative period and quantitative analysis of the EZ disruption have been reported only in a few studies [9, 10, 15, 16].

In this study, we used SS-OCT for pre- and postoperative examinations of eyes with surgically closed idiopathic MH. We analyzed 3D-scan images and evaluated the restoration rate for EZ disruption and the diameter of residual EZ disruption at 1 year after surgery. EZ was restored in 53% of eyes and the mean residual EZ diameter was 54.7 ± 33.1 *μ*m. Whether EZ was restored or not did not affect logMAR VA at 1 year after surgery.

Michalewska et al. reported that only 29.5% of eyes had photoreceptor defects 12 months after MH surgery and the mean size of the photoreceptor layer defect was 60 *μ*m [15]. In their study, 50 horizontal OCT scans were performed around the macula. Each scan had an interval of 140 *μ*m, which was larger than the mean size of the photoreceptor layer defect they observed. Postoperative photoreceptor layer defects might remain in more eyes than reported because some defects smaller than the scan interval might have gone undetected. Bottoni et al. used raster scanning with the scan interval ranging from 119 to 252 *μ*m; they reported that 52.6% of eyes had a continuous EZ at 12 months [12]. Our results had higher resolution than these studies. In our study, 3D-scan had a scan interval of 23.44 *μ*m, which was less than half of the mean diameter of the residual EZ disruption. However, it is possible that EZ disruption smaller than 23.44 *μ*m was missed to be identified in our study. Our results do not indicate complete restoration of EZ disruption in Group A, as suggested in a previous study [16, 18]. Ooto et al. used adaptive optics scanning laser ophthalmoscopy (AO SLO) and showed the presence of dark areas in the photoreceptor layer (0.004 to 0.754 mm^2^) in 21 of 21 eyes after anatomically successful MH closure [19].

We investigated both the EZ restoration rate and the diameter of the residual EZ disruption at 1 year after surgery, which have not been analyzed simultaneously. Wakabayashi et al. reported that EZ restoration was observed in 50% of eyes 12 months postoperatively [10]. Kang et al. reported that outer foveolar defects, which were identified on the initial OCT after surgery for MH, disappeared in 37 of 44 eyes during the 4 weeks to 18 months after surgery [16]. In these studies, the size of the residual EZ disruption was not analyzed. Chen et al. measured the EZ defect diameter pre- and postoperatively, but their follow-up period was limited to only 12 weeks [20]. Inoue et al. used a scan with 200 lines across a 6 mm square (with an interscan interval of 30 *μ*m) to measure the length of the EZ defect 12 months after MH surgery [9]. In their study, the EZ restoration rate was not shown.

We included only eyes that had both EZ disruption and a continuous ELM during the early postoperative period (within 10 days) (Figure 1(a)). Wakabayashi et al. classified the status of the photoreceptor layer into 3 groups [10]. The first group had restoration of both of the EZ and the ELM. The second group had disruption of the EZ but a restored ELM. The third group had disruption of both of the EZ and the ELM. Eyes in their second group corresponded to the eyes included in our study. In the early postoperative period, there were no eyes in their first group.

We excluded eyes in the third group (Figure 1(b)) because the eyes of this group had both ELM and EZ disruption, and the process of EZ restoration is different or complicated compared to that of the second group. In a previous clinicopathologic study, Funata et al. reported a case of surgically closed stage 3 MH with the replacement of hole by glial cell proliferation [21]. This glial proliferation is considered to be represented as foveal moderate-reflective or hyperreflective lesion on OCT images [10, 12, 17]. Wakabayashi et al. reported that all eyes corresponding to the third group had a hyperreflective lesion replacing all intraretinal layers at the fovea and it remained 12 months after surgery [10]. The case with glial cell proliferation reported by Funata et al. had underwent vitrectomy 3 years before autopsy [21]. In contrast, Bottoni et al. reported that a moderately reflective lesion partially or completely replaced the normal foveal architecture after hole closure but it regressed during 12-month follow-up in some eyes [12]. The eyes of the second and third groups should be investigated separately for precise analysis of EZ restoration.

LogMAR VA at 1 year after surgery was significantly improved compared to preoperative VA in each group (Group A, 0.592 ± 0.243 to −0.017 ± 0.102; *P* < 0.001; Group B, 0.610 ± 0.343 to 0.051 ± 0.148; *P* < 0.001) (Table 3). Both groups had very good VA and there was no significant difference found between them. Our inclusion criteria, EZ disruption and a continuous ELM found on OCT during the early postoperative period, may predict good visual prognosis in eyes with surgically closed MH, regardless of the status of the EZ disruption during the late postoperative period.

Significant correlation was not found between logMAR VA and the diameter of the EZ disruption at 1 month, 3 months, 6 months, and 1 year after surgery in our study. In contrast, Ooka et al. reported significant correlations between the length of the EZ defect, best corrected visual acuity, and foveal sensitivity at 1 month, 3 months, and 6 months after surgery [11]. In our study, the postoperative mean logMAR VA was better and the mean diameter of EZ disruption was much smaller than their study at each period. The mean preoperative MH diameter in our study was also smaller than their study. We consider that these differences were caused by the difference of inclusion criteria of subjects. The distributions of logMAR VA and EZ disruption diameter were restricted to a very narrow range from an early stage after the surgery and a significant correlation between them was not observed in our study (Figure 3).

EZ restoration was not significantly associated with differences in visual prognosis at 1 year after surgery. Based on these results, it is possible that a residual EZ disruption smaller than 60 *μ*m in diameter does not affect VA in eyes with surgically closed MH. Wakabayashi et al. reported that BCVA did not significantly differ in groups that corresponded to our Groups A and B at 12 months after surgery [10]. Ruiz-Moreno et al. used the same grouping criteria as Wakabayashi et al. and reported similar results [22]. A diameter of 60 *μ*m in the retina is equivalent to a visual angle of approximately 10 min of arc. It is same size as size II spot with standard Goldmann perimetry, which is not considered small in the central visual field. Visual sensitivity loss or focal metamorphopsia must exist around a residual EZ disruption. Further studies with ophthalmic parameters other than VA should be considered to evaluate the effect of EZ disruption more precisely in eyes with surgically closed MH.

## 5. Conclusions

EZ disruption was restored in 53% and remained in 47% of eyes at 1 year after surgical closure of idiopathic MH that had both EZ disruption and a continuous ELM during the early postoperative period. Mean residual EZ disruption diameter was 54.7 ± 33.1 *μ*m. LogMAR VA at 1 year after surgery was significantly better than preoperative VA in each group and significant difference was not found between them. Whether EZ was restored or not did not influence logMAR VA at 1 year after surgery.

## Figures and Tables

**Table 1:** Characteristics of two groups.

	Group A *n* = 10	Group B *n* = 9	Difference between Groups A and B
Age (years)	55–79 65.1 ± 7.6	51–65 62.4 ± 4.4	*P* = 0.377^*∗*^

Duration of symptoms (months)	1–9 3.10 ± 2.42	1–5 2.78 ± 1.20	*P* = 0.723^*∗*^

Preoperative MH diameter (*μ*m)	70–516 307.2 ± 155.0	188–789 370.4 ± 188.5	*P* = 0.432^*∗*^

Stage 2/3/4 (number of eyes)	4/5/1	2/2/5	*P* = 0.0573^†^

MH: macular hole.

^*∗*^Student's *t*-test.

^†^Difference in the number of stage 4 holes; Fisher's exact probability test.

**Table 2:** Diameter of EZ disruption.

	Group A *n* = 10	Group B *n* = 9	Difference between Groups A and B
Postoperative 1 month (*μ*m)	0–187.5 96.2 ± 65.9	70.3–375.0 234.4 ± 125.5	*P* = 0.009^*∗*^

Postoperative 3 months (*μ*m)	0–187.5 38.0 ± 57.5	23.4–257.8 125.0 ± 99.1	*P* = 0.041^*∗*^

Postoperative 6 months (*μ*m)	0–117.2 25.8 ± 37.4	23.4–234.4 89.9 ± 74.7	*P* = 0.037^*∗*^

Postoperative 1 year (*μ*m)	0 0	23.4–140.6 54.7 ± 33.1	*P* < 0.001^*∗*^

EZ: ellipsoid zone.

^*∗*^Student's *t*-test.

**Table 3:** Preoperative and postoperative logMAR VA.

	Group A *n* = 10	Group B *n* = 9	All subjects *n* = 19	Difference between Groups A and B
Preoperative	0.155–1.000	0.097–1.046		*P* = 0.894^*∗*^
	0.592 ± 0.243	0.610 ± 0.343	0.600 ± 0.286	

Postoperative 1 month	−0.079–0.523	0.000–0.523		*P* = 0.527^*∗*^
	0.188 ± 0.246	0.255 ± 0.183	0.218 ± 0.217	
	(*P* < 0.001^#^)	(*P* = 0.019^#^)	(*P* < 0.001^#^)	

Postoperative 3 months	−0.079–0.398	0.000–0.398		*P* = 0.451^*∗*^
	0.078 ± 0.172	0.137 ± 0.144	0.104 ± 0.158	
	(*P* < 0.001^#^)	(*P* = 0.002^#^)	(*P* < 0.001^#^)	

Postoperative 6 months	−0.079–0.301	−0.079–0.222		*P* = 0.750^*∗*^
	0.061 ± 0.146	0.083 ± 0.128	0.070 ± 0.135	
	(*P* < 0.001^#^)	(*P* = 0.003^#^)	(*P* < 0.001^#^)	

Postoperative 1 year	−0.079–0.155	−0.079–0.301		*P* = 0.332^*∗*^
	−0.007 ± 0.102	0.051 ± 0.148	0.021 ± 0.126	
	(*P* < 0.001^#^)	(*P* < 0.001^#^)	(*P* < 0.001^#^)	

^*∗*^Student's *t*-test.

^#^Comparison with preoperative logMAR VA; paired *t*-test.
